# Minimally invasive peritoneal dialysis catheter insertions with concomitant cholecystectomy or hernioplasty: Surgical technique and our experiences

**DOI:** 10.1177/11297298211039447

**Published:** 2021-08-16

**Authors:** Jurij Janež, Armand Dominik Škapin

**Affiliations:** 1 Department of Abdominal Surgery, University Medical Centre Ljubljana, Ljubljana, Slovenia; 2 University of Ljubljana, Faculty of Medicine, Ljubljana, Slovenia

**Keywords:** Laparoscopy, peritoneal dialysis catheter insertion, concomitant procedure, cholecystectomy, hernioplasty

## Abstract

**Introduction:**

Peritoneal dialysis is a well-accepted replacement therapy in patients with end-stage renal disease. There are many different options adopted on how to insert a peritoneal dialysis catheter. In our institution, a laparoscopic insertion has become the method of choice for providing peritoneal dialysis access in adult patients. The aim of this study was to analyze surgical outcomes of patients after laparoscopically assisted placement of a PD catheter some of them after concomitant cholecystectomy or hernioplasty.

**Methods:**

We have evaluated 70 consecutive patients from 1st of October 2015 to 30th of April 2020 who underwent laparoscopic insertion of a peritoneal dialysis catheter. Demographic data, details about surgery and about peri- and postoperative complications were gathered.

**Results:**

Out of 70 enrolled patients, 15 had gallstones (21%) and underwent concomitant laparoscopic cholecystectomy. Three patients (4%) had abdominal wall hernia and underwent concomitant hernioplasty. We observed no perioperative complications connected with any of the performed procedures. There was one early postoperative complication: an early leak of dialysate fluid. Late complications were observed in nine patients (13%): mechanical catheter problems (two patients), peritonitis (three patients), skin exit-site infections (two patients), peri-catheter leak (one patient), and port-site hernia (one patient).

**Conclusions:**

For all patients with concomitant gallbladder disease or abdominal wall hernias we suggest to perform synchronous surgeries, due to finding no more complications after concomitant procedures in comparison to patients in whom only a PD catheter was inserted. Concomitant procedures are done to spare patients two separate procedures and to avoid possible complications. We also suggest using the cholecystectomy first, PD catheter insertion second approach for having excellent peri- and postoperative results.

## Introduction

Peritoneal dialysis (PD) is one of the three methods for treatment of patients with end-stage renal disease (ESRD). The other two options are hemodialysis (HD) and kidney transplantation. Worldwide more than 10% of patients with ESRD are treated with PD, in our country only 2%–3% are.^1^ For PD to be effective, it is very important to provide a quality PD access with an insertion of a functional and durable PD catheter.^2^ Besides the infectious complications, mechanical catheter problems are the most important cause for PD catheter failure. There are several different ways to insert a PD catheter: open surgical, laparoscopic, peritoneoscopic, blind percutaneous, and ultrasound-guided percutaneous technique.^3,4^ In our institution, a laparoscopically assisted insertion of a PD catheter has become the standard method for providing PD access in adult patients with ESRD. Patients with concomitant abdominal wall hernias can develop complications after peritoneal dialysis is started and should undergo hernioplasty prior. Likewise, patients with gallbladder disease should undergo cholecystectomy to avoid possible infections, compromising the catheter and a potential transplanted kidney. Therefore, it would make sense to perform synchronous procedures when indicated which is not an establish practice yet. In this article we present our experience and surgical outcomes of seventy consecutive patients with ESRD, who had a laparoscopically assisted placement of a PD catheter, some of them with concomitant cholecystectomy or hernioplasty.

## Methods

We have performed a retrospective analysis of 70 consecutive patients, who had a laparoscopic insertion of a PD catheter from 1st of October 2015 to 30th of April 2020. The analysis was done according to the patients’ charts. Regular follow-ups were every 3 months for all patients as long as the catheter was inserted. The median follow-up time was 3 years. In all patients, PD catheters were used for dialysis 4–6 weeks after insertion. We have collected demographic data (age, gender, reason for ESRD, comorbidities, previous surgeries, previous treatment with PD or HD), data about surgery (concomitant procedures, other complications during surgery), early postoperative complications within 30 days from surgery (infections, bleeding, PD catheter problems, early peri-catheter leaks), and late complications (PD catheter problems, infections, port site hernias, removal of PD catheters, transfer to HD, peri-catheter leaks). All included patients gave written consent to participate.

## Surgical technique

**Figure 1.**
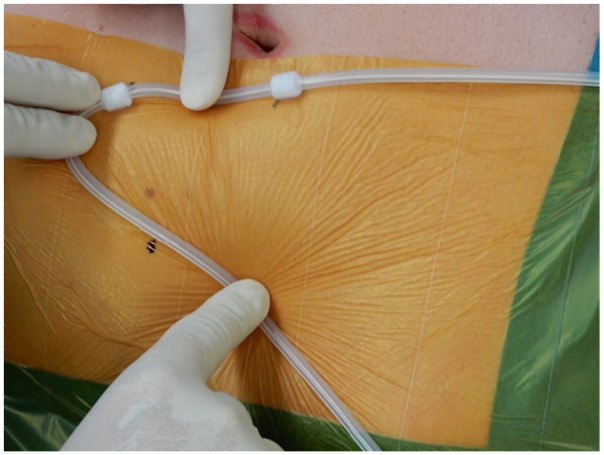
Marking the positions of a deep cuff, subcutaneous cuff, and the skin exit site prior to incision. Source: Personal.

**Figure 2.**
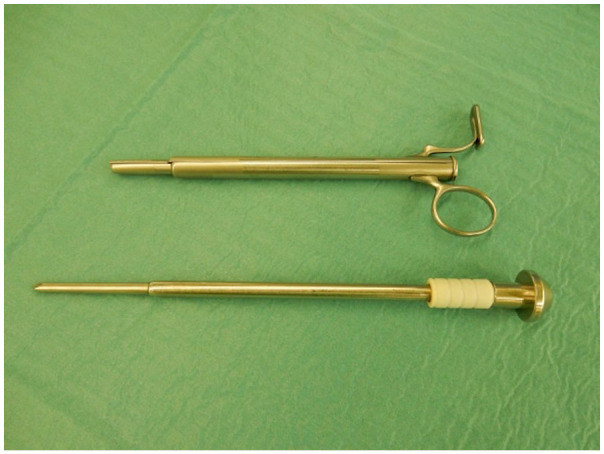
A special trocar, patented by Zoran Cala, used for rectus sheath tunneling. Source: Personal.

**Figure 3.**
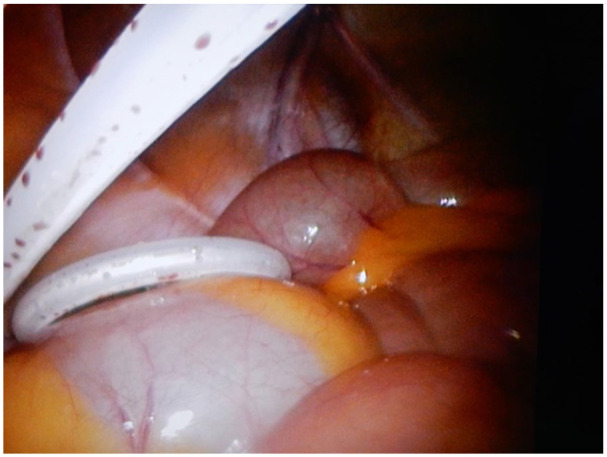
Intraoperative view of inserted PD catheter. Source: Personal.

The surgical technique for PD catheter insertions was in all cases laparoscopically assisted technique. All procedures were performed by one experienced abdominal surgeon using the same equipment. PD catheters used were coiled Tenckhoff catheters with two Dacron cuffs. Prior to incision, positions of deep cuff, subcutaneous cuff and skin exit site were marked with a sterile pencil on the anterior abdominal wall (coiled tip of the catheter is aligned with the upper border of the pubic symphysis, the deep cuff is positioned paraumbilically—approximately 1 cm lateral to umbilicus, next the subcutaneous cuff is positioned cranially, somewhat more lateral, and then the catheter is rotated caudally; skin exit site is marked approximately 4 cm along the catheter from the subcutaneous cuff) (Figure 1). Surgery began with laparoscopy through a supraumbilical approach by creation of pneumoperitoneum with Veress needle and insertion of a 5 mm non-bladed, optical trocar. We used two 5 mm optical trocars (one for camera and the other for a laparoscopic grasper, needed for proper positioning of a catheter tip) and a special trocar for rectus sheath tunneling patented by Cala^5^ (Figure 2). This trocar was inserted through a paraumbilical skin incision, passed obliquely through the abdominal wall and directed into the pelvis. The insertion was done under laparoscopic monitoring to avoid any injury of abdominal organs or inferior epigastric vessels. This way we created a canal that was long enough for the part of the catheter between the two cuffs. The PD catheter was then introduced through the trocar and the coiled tip was placed in the pelvic region (Figure 3). Skin exit site was determined on the left or right side of the anterior abdominal wall.

In cases, when a concomitant procedure had to be performed, such as cholecystectomy or hernia repair, some steps in the whole procedure were somewhat modified. In cases of concomitant cholecystectomy, we used “cholecystectomy first, PD catheter insertion second” approach. The PD catheter was inserted only after the gallbladder had already been removed from the abdominal cavity. In these cases, an 11 mm supraumbilical optical trocar was used. For working instruments, an 11 mm epigastric optical trocar and two 5 mm optical trocars under the right costal margin were used. Inguinal hernias were repaired with prosthetic mesh according to Lichtenstein technique before the PD catheter was inserted and the sole umbilical hernia was repaired with direct sutures (because the fascial defect was only 1 cm in diameter) after the PD catheter had already been inserted.

## Results

In the observed time frame, we have performed 70 laparoscopic insertions of PD catheters. Demographic data of the observed patients is presented in Table 1. Fifteen patients (21%) underwent concomitant laparoscopic cholecystectomy due to gallstones, two patients (3%) had concomitant inguinal hernia repair (one left and one right), and one patient (1%) had concomitant umbilical hernia repair. Altogether, 18 patients (26%) underwent concomitant procedures.

**Table 1.** Demographic data of patients.

	Number of patients = 70
Gender	
Male	41
Female	29
Average age at operation	70 (min 16–max 87)
Reason of ESRD	
Transplanted kidney failure	13
IgA glomerulonephritis	31
Alport syndrome	14
Diabetic nephropathy	8
Polycystic disease	2
Hypertensive nephropathy	2
Comorbidities	
Diabetes mellitus	8
Arterial hypertension	11
Hyperlipidemia	11
Cholecystolithiasis	15
Abdominal wall hernias	3
Other	12
Previous operative procedures	
Kidney transplantation	12
Cholecystectomy	1
Hernia repair	1
Appendectomy	2
Suturing of perforated gastric ulcer	1
Previous PD catheter insertion	2
Previous treatment with HD	25

There were no major nor minor complications during any of the surgeries. There was one early postoperative complication (within 30 days from surgery). It was an early leak of dialysate fluid in the subcutaneous tissue—the PD catheter was removed and the patient was thereafter treated with hemodialysis.

Late complications (more than 30 days after surgery) are presented in Table 2. Two patients had PD catheter obstruction. In one of them the catheter tip migrated and was wrapped with omentum. The patient underwent laparoscopy; partial omentectomy was performed and the catheter tip was placed back into the pelvic region. In the other patient, the catheter tip was found to be in proper position, but was wrapped with fimbriae. Therefore, laparoscopic lysis was performed. Three patients developed resistant peritonitis and had PD catheters consequently removed. Catheter was also removed from one patient with skin exit-site infection and from one patient with late peri-catheter leak. The other patient with skin exit-site infection had a new catheter inserted on the other side of the abdomen. Altogether, seven catheters (10%) got removed due to postoperative complications. One patient developed an umbilical port-site hernia, which was repaired with direct sutures.

**Table 2.** Late complications (more than 30 days after surgery).

Type of complication	Number of patients	Time from operation to onset of complication
Catheter obstruction	1	3 months
Catheter tip dislocation and obstruction	1	6 months
Peritonitis	3	All more than 6 months
Skin exit-site infection	2	Both 3 months
Peri-catheter leak	1	More than 6 months
Umbilical port-site hernia	1	6 months

In all 18 patients with concomitant procedures, we did not observe any peri- or postoperative complications connected with neither PD catheter insertions nor concomitant procedures.

## Discussion

With the development of minimally invasive surgery, the laparoscopic insertion of PD catheters is gaining popularity worldwide.^6^ One of the advantages is the possibility of diagnostic laparoscopy which is performed to evaluate the abdominal cavity for possible pathologies that can be resolved prior to catheter insertion. Special attention is made to the pelvic region, where the catheter tip will be placed under direct vision.

An important aspect of laparoscopic insertions is also the employment of minimal number of small trocars, in order to avoid port-site leaks of dialysate fluid and possibly the development of hernias.^7^ The use of non-bladed, optical trocars further lowers the incidence of trocar-site hernias.^8^ In the series, we have always inserted one laparoscopic port in the midline, supraumbilically. Some authors are of the opinion that midline insertion of trocars comes with a greater occurrence of trocar site hernias compared to lateral insertion. However, Tonouchi et al.^9^ have come to a conclusion that the incidence of trocar site hernias in the lateral region is no less than in the midline. Furthermore, in the enrolled patients we have observed only one umbilical trocar-site hernia formation. Nevertheless, fascia at port-sites should be closed with sutures in order to prevent these complications.

Maheshwari^6^ recommend routine omentopexy, especially when the omentum is long enough to reach the catheter in its final position . Omentopexy is preferred over omentectomy because it is quicker and does not carry the same risk of postoperative hemorrhage. In the observed patients, however, we did not perform routine omentopexy and only in one case did the omentum wrap around the catheter and obstruct it. According to our experience, we believe the routine omentopexy or omentectomy isn’t necessary in most cases. It is, however, reasonable in select cases, where omentum is very long and falls deep into the pelvis.

Quanquan et al. recommend catheter fixation to parietal peritoneum or to intra-abdominal organ such as uterus or bladder to prevent catheter tip migration.^10^ Suturing the catheter to a pelvic structure is, however, not the best practice due to possible erosion of the suture with subsequent catheter migration, difficulty removing the catheter at a later date and it was even found to be a predictor of catheter failure.^11^ In the enrolled patients we didn’t perform routine catheter fixation, and we have observed only one catheter tip migration from pelvic region. When the rectus sheath tunneling is performed properly, the PD catheter is oriented toward the pelvis and both cuffs are placed in the correct positions (deep cuff preperitoneally and outer cuff in the subcutaneous tissue), the tissue fibrosis around cuffs provides sufficient fixation of the catheter. In such cases there is no need for additional internal or external fixation.^12,13^ Rectus sheath tunneling effectively keeps the catheter oriented toward the pelvis, eliminates the peri-catheter hernias, and reduces the risk of peri-catheter leaks.^14^

When inserting a PD catheter, latest guidelines recommend patients with gallstones/gallbladder polyps or abdominal wall hernias undergo concomitant gallbladder removal or hernia repair.^14–16^ Fifteen of the enrolled patients had gallstones and 3 had abdominal wall hernias.

All patients with gallstones underwent concomitant laparoscopic cholecystectomy. We used the “cholecystectomy first, PD catheter insertion second” approach. Crabtree et al.^16^ are, however, concerned with this approach and recommend “clean procedure first (catheter placement) and clean-contaminated procedure second (cholecystectomy)” approach. Possible reason for preferring a different sequence according to some authors is a decreased probability of infection. When performing cholecystectomy there is always a possibility of inadvertent bile spillage. Even with asymptomatic biliary tract disease, the bile is not always sterile and may include anaerobes.^17^ Consequently, bacteria can contaminate transmural course during subsequent PD catheter insertion (relatively avascular fatty tissue) and with the presence of a foreign body (the catheter), the threshold for infection is lowered. Performing the catheter insertion first and applying dressings to cover the closed wounds would minimize the contamination. However, we did not adopt this recommendation for having excellent experience with our own approach. When performing cholecystectomy first, it is easier to achieve a meticulous hemostasis, to collect all the gallstones, to wash the abdominal cavity, and in case of any complications, the PD catheter doesn’t need to be inserted, saving it from a likely failure. In our approach it is, therefore, very important to perform the cholecystectomy as clean as possible, without perforation and with the use of laparoscopic specimen bag. If all those conditions are met then the cholecystectomy can be considered clean and, according to our experiences, does not present any survival threat for a subsequently inserted PD catheter. In all 15 patients we did not observe any peri- or postoperative complications. On the other hand, even elective cholecystectomy performed after a PD catheter had already been inserted can become complicated. In such cases, the already inserted PD catheter would become contaminated and its survival could also be compromised. Either way, although not stated in the latest ISPD (International Society for Peritoneal Dialysis) guidelines,^16^ it would make sense to add anaerobic antibiotic coverage (e.g. metronidazole) to the usual anti-staphylococcal antibiotic prophylaxis when concomitant cholecystectomy is planned regardless of the sequence of the procedures.

Some authors recommend concomitant cholecystectomy only in symptomatic biliary tract disease; however, our national renal transplant program insists on performing cholecystectomy even in patients with asymptomatic gallbladder disease prior to being listed for renal transplantation.^16,18–21^ And with a concomitant procedure, it is not needed to be performed later. Also, asymptomatic biliary tract disease can become symptomatic after the patient has already had the PD catheter inserted requiring cholecystectomy, which would unnecessarily threaten the catheter’s survival.

Abdominal wall hernias should be repaired prior to or at the time of PD catheter insertion, because otherwise the dialysate fluid is sequestered in the hernia sac causing unpredictable dialysis clearance and ultrafiltration.^18,19^ Furthermore, peritoneal dialysis was found to increase the risk of abdominal hernia formation and can cause progressive enlargement of already existing hernia sacs.^22^ For all those reasons, early surgical repair has been advocated. Laparoscopic hernioplasty is not recommended in patients with an already inserted PD catheter, because the disruption of peritoneum in laparoscopic hernia repair can cause peritoneal leaks and can also alter the PD catheter function.^20^ In such cases open hernioplasty is preferred. All three patients with abdominal wall hernias underwent hernioplasty at the time of the PD catheter insertion. The umbilical hernia was repaired with direct sutures and not with prosthetic mesh because the fascial defect was only 1 cm in diameter as is in accordance with the newest guidelines reported by the European and the Americas Hernia Society.^23,24^ We did not observe any peri- or postoperative complications connected with either of the two procedures.

With this study we have demonstrated that it is safe to perform concomitant procedures when indicated. Also, adopting cholecystectomy first approach showed excellent results in our patients.

The advantages of our research were that there was only one surgeon performing all of the procedures and that all patients were treated by the same principles. On the other hand, having only one surgeon in a study lowers the generalizability of the findings. Limitations include a relatively low number of enrolled patients, especially the ones after concomitant procedures and it being a single-center study.

## Conclusions

In our institution, a laparoscopic placement of a PD catheter has become the method of choice for providing peritoneal dialysis access in adult patients. Analysis of surgical outcomes of 70 patients who underwent laparoscopically assisted placement of a PD catheter showed no major nor minor perioperative or early postoperative complications. Patients with gallstones/gallbladder polyps or abdominal wall hernias underwent concomitant procedures which is in accordance with the latest guidelines. After concomitant procedures patients didn’t have any peri- or postoperative complications connected with either of the surgeries. This approach spares the patient two or more separate procedures and has an overall lower risk of PD catheter failure. Therefore, we suggest patients undergo concomitant procedure when indicated, and to use the cholecystectomy first approach in patients with conjoined gallbladder disease.
